# Sustainable Micro and Nano Additives for Controlling the Migration of a Biobased Plasticizer from PLA-Based Flexible Films

**DOI:** 10.3390/polym12061366

**Published:** 2020-06-17

**Authors:** Laura Aliotta, Alessandro Vannozzi, Luca Panariello, Vito Gigante, Maria-Beatrice Coltelli, Andrea Lazzeri

**Affiliations:** 1 Department of Civil and Industrial Engineering, University of Pisa, 56122 Pisa, Italy; alessandrovannozzi91@hotmail.it (A.V.); luca.panariello@ing.unipi.it (L.P.); vito.gigante@dici.unipi.it (V.G.); andrea.lazzeri@unipi.it (A.L.); 2 Consorzio Interuniversitario Nazionale per la Scienza e Tecnologia dei Materiali (INSTM), 50121 Florence, Italy

**Keywords:** poly(lactic acid), poly(butylene succinate), plasticizer migration, diffusion

## Abstract

Plasticized poly(lactic acid) (PLA)/poly(butylene succinate) (PBS) blend-based films containing chitin nanofibrils (CN) and calcium carbonate were prepared by extrusion and compression molding. On the basis of previous studies, processability was controlled by the use of a few percent of a commercial acrylic copolymer acting as melt strength enhancer and calcium carbonate. Furthermore, acetyl n-tributyl citrate (ATBC), a renewable and biodegradable plasticizer (notoriously adopted in PLA based products) was added to facilitate not only the processability but also to increase the mechanical flexibility and toughness. However, during the storage of these films, a partial loss of plasticizer was observed. The consequence of this is not only correlated to the change of the mechanical properties making the films more rigid but also to the crystallization and development of surficial oiliness. The effect of the addition of calcium carbonate (nanometric and micrometric) and natural nanofibers (chitin nanofibrils) to reduce/control the plasticizer migration was investigated. The prediction of plasticizer migration from the films’ core to the external surface was carried out and the diffusion coefficients, obtained by regression of the experimental migration data plotted as the square root of time, were evaluated for different blends compositions. The results of the diffusion coefficients, obtained thanks to migration tests, showed that the CN can slow the plasticizer migration. However, the best result was achieved with micrometric calcium carbonate while nanometric calcium carbonate results were less effective due to favoring of some bio polyesters’ chain scission. The use of both micrometric calcium carbonate and CN was counterproductive due to the agglomeration phenomena that were observed.

## 1. Introduction

The environmental impact of plastic wastes, also due to the limited disposal methods, is a continuously growing public concern worldwide. This problem has encouraged research and industrial interest on biobased and biodegradable polymers that could overcome sustainability issues and environmental challenges posed by the production and disposal of oil-derived plastics [1,2]. In packaging and agriculture applications, the use of bio-based biodegradable polymers is a strong advantage both for environment and customers [3]. However, it is expected that the demand for these biopolymers will increase and, over the coming few decades, bioplastic materials can complement and gradually substitute the oil-based plastics in different sectors [2,4]. Nowadays different biopolymers can be found not only in food and agriculture applications, but also in the medicine and cosmetic sectors. The development of renewable polymeric modulated materials tailored for specific applications is a subject of active research interest worldwide [5].

Poly(lactic acid) (PLA) is particularly interesting because it exhibits mechanical properties (Young’s modulus of about 3 GPa, tensile strength between 50 and 70 MPa with an elongation at break of around 4%, and an impact strength close to 2.5 kJ/m^2^) that make it useful for a wide range of applications [6,7]. It can be obtained from renewable resources (e.g., corn, wheat, or rice) and it is not only biodegradable and compostable, but also recyclable. However, in particular, its biocompatibility makes it appealing for biomedical and cosmetic applications. If compared to other commercialized biopolymers (such as poly(hydroxylalkanoates) (PHAs), poly(caprolactone) (PCL), and starch), PLA is easily processable [8]. However, PLA’s brittleness and its poor heat resistance limit strongly the application of PLA. The improvement of PLA’s toughness can be reached in different ways (that can also be adopted contemporary): plasticization, copolymerization, and melt blending with flexible polymers [9,10]. However, the blending technique is the common method adopted to overcome the PLA brittleness making it useful in those applications (like film production) where high flexibility and toughness are requested. In literature different successful studies can be found where PLA was blended, in different quantities, with other biodegradable polymers such as: polycaprolactone [11], poly(vinyl alcohol) (PVOH) [12,13], polybutylene(succinate) (PBS) [14,15,16], poly(butylene succinate-co-adipate) [17,18,19], and poly(butylene adipate-co-terephthalate) (PBAT) [20,21,22].

Furthermore, the combination of additives such as plasticizers into biopolymers and their blends, is a common practice to further improve the mechanical flexibility, and processability limitations. Generally, plasticizers are a class of low-to-medium molecular weight compounds up to a few thousand, it is expected that their demand will increase reaching approximately the 9.75 million of tons in 2024 [23]. In various applications, like film formation or coating dispersion, the adequate selection of plasticizer greatly improves the processing; however, the choice of these plasticizers, especially in biobased applications, is limited by the required safety, environmental favorability, and chemical and physical properties that dictate their miscibility [2].

It is therefore evident that, unlike classic plastic commodities (e.g., HDPE) which possess good starting physical-chemical properties, biopolyesters need to be improved and in this context biopolymers can release a major amount of additives that are not covalently bound to the polymer, at all stages of the plastic’s lifecycle via migration of liquids or solids or via volatilization [24]. The result of this release is the transfer of chemicals (such as plasticizers) that can affect human health and can contaminate soils and water [25,26]. In fact, especially in natural environments, liquid additives can quickly migrate out of plastic and can be absorbed by roots affecting the plant development [27]. On the other hand, a controlled release of beneficial substances contained in the blends are very useful in the cosmetics and biomedical sectors [28,29].

For all of these aspects, the study of migration is very important especially for films containing plasticizers that are not chemically attached to polymeric chains (not reactive plasticizers). Depending on certain conditions, liquid plasticizers can come out from the polymer matrix. During service and storage, this loss is problematic because it leads to unwanted changes of the mechanical properties (loss of flexibility and toughness with an increment of stiffness). Clearly, this decrement of the mechanical properties will be more evident and dangerous in those polymeric matrices that are brittle in the not plasticized state (like PLA). This plasticizer release, as well as creating problems of stiffening, can lead to variations in the crystallization of the samples due to the reorganization over time (aging) of the crystalline structures [30]. Even the plasticizer molecular weight and linearity of the plasticizer influence the migration and so an average plasticizer molecular weight is required to ensure long-term plasticizer retention in the polymeric matrix [31].

According to the type of plasticizer, different strategies can be adopted to prevent the plasticizer migration (such as internal plasticization [32], polymer surface modification [33,34], or addition of nanofillers or ionic liquid [35,36]). The methodologies and the advantages/disadvantages of these strategies are extensively reported in literature [37]. Among the strategies mentioned, the role of micro and nano filler addition on the plasticizer release is particularly interesting.

In fact, the use of fillers has a double effect: they can reduce plasticizer migration thanks to the creation of tortuosity that forces the plasticizer molecules to follow a longer path to leave the polymeric structure [38] and the adsorption mechanism. Fillers, especially nanoparticles or nanofibrils, are extremely small and they possess a large number of groups capable of interactions on their surfaces that make the absorption of other substances easy. On the basis of these characteristics, the plasticizer migration can be reduced both by the absorption on the surface of plasticizer molecules and by the steric resistance that makes the passage of the plasticizer difficult [37]. On the other hand, filler addition can improve the mechanical properties of the materials (such as toughness) according to the rigid filler toughening mechanism [39]. Various nano-scale and micro-scale fillers with different geometries (such as: montmorillonite, silica, calcium carbonate, and aluminum oxide) are reported to improve not only the properties of polymers such as toughness, stiffness, and heat resistance [40,41] but they also can limit the plasticizer migration [35,38]. For example, it has been observed that organic montmorillonite (OMMT), nano-SiO_2_, and nano-CaCO_3_ have a strong adsorption force and diffusion inhibition [35,37]. Therefore, the presence on the fillers surface of functional groups which are active and can easily bond with plasticizer molecules is another important aspect that influences the migration of the plasticizer [42]. It was observed that the migration rates decrease with the increasing of filler content [43]. However, it is essential to reach a homogeneous dispersion of the particles in the polymeric matrix to hinder the plasticizer migration. In fact, the increase of fillers content does not lead always to better barrier properties if there is a poor dispersion of nano particles in the matrix [35]. Clearly, great attention must be paid to the combination of polymeric plasticizer and micro/nano additives to control the eventual plasticizer migration and at the same time to reach an optimal combination of mechanical properties (good balance between stiffness and toughness).

Understanding the mechanism and the kinetics of plasticizer loss is fundamental also to evaluating the short- and long-term performance of the final plasticized material. For instance, if the correlation between mechanical properties and plasticizer concentration is known, it will be possible to predict the change of mechanical properties and, consequently, the lifetime of products. Furthermore, it will be also possible to develop new methods to eliminate/control the plasticizer migration.

In this paper, the attention is focused on the results of previous studies where suitable biobased skin compatible films were successfully prepared and investigated. Different additives were added: calcium carbonate, chitin nanofibrils as functional filler (dispersed by poly(ethylene glycol) (PEG)), melt strength enhancer (Plastistrength), and acetyl tributyl citrate (ATBC) plasticizer. The addition of all these additives has led to an improvement of the melt processability, of the mechanical properties (the films resulted flexible and high resistant) and of the antimicrobial and anti-inflammatory properties [44,45,46,47]. However, troubles were encountered during the storage of films where 4 wt % of micrometric calcium carbonate was present [44] due to the plasticizer migration. Interestingly, any data were reported about the effect on plasticizer migration of natural nanofibers obtained by sea food waste, chitin nanofibrils used in these bionanocomposites for their indirect anti-microbial activity [47].

In the present paper, the identification of the diffusion coefficients for the examined blends was carried out. Furthermore, in order to limit and/or to control the plasticizer migration, different types of commercial calcium carbonate particles (micro and nanometric) and chitin nanofibrils were used. The effect of their addition on the films processability and on the mechanical properties was carried out to ensure that no significant variations occurs in the already optimized rheological and mechanical properties. Finally, the effect of the introduction of CaCO_3_ and chitin nanofibrils on the ATBC migration was evaluated through the diffusion coefficient calculation. The diffusion coefficients were obtained, applying analytical correlations based on the Fick’s second law, by regression of the experimental migration data plotted as the square root of time.

## 2. Materials and Methods

### 2.1. Materials

The polymeric granules and additives used in this work for the blend’s preparation are:

- Commercial poly(lactic) acid (PLA), trade name 2003D, produced by Nature Works LLC (Minnetonka, Minneapolis, MN, USA) was used. This is a commercial grade containing about 4% of D-lactic acid units that lower the melting point and the crystallization tendency, improving the processing ability. This PLA, according to the producer’s data sheet has a density of 1.24 g/cm^3^, a melt flow index (MFI) of 6 g/10 min (210 °C, 2.16 kg) and a nominal average molar mass of 200,000 g/mol.
- Poly(butylene succinate) (PBS), trade name BioPBS FD92PM, purchased from Mitsubishi Chemical Corporation (Tokyo, Japan). It is a copolymer of succinic acid, adipic acid and 1,4-butandiol with a melt flow index (MFI) of 4 g/10 min (190 °C, 2.16 kg) and a density of 1.24 g/cm^3^.
- Acetyl tributyl citrate (ATBC), a product of Tecnosintesi S.p.A (Bergamo, Italy), was used as biobased and biodegradable plasticizer. It is a colorless and odorless liquid having a density of 1.05 g/cm^3^ and a molecular weight of 402.5 g/mol.
- Plastistrength 550 (named PST for brevity), commercialized from Arkema (Paris, France), is a medium molecular-weight acrylic copolymer that appears as a white powder with a density of 1.17 g/cm^3^. It is a commercial melt strength enhancer commonly added to improve the melt processability.
- Poly(ethylene glycol) (PEG6000) provided by Sigma-Aldrich (St. Louis, MO, USA) was used, for improving the dispersion of chitin nanofibrils [48]. It is a colorless solid, with a high molecular weight of 6000 g/mol and a solubility in water of 50 mg/mL at 20 °C.
- Chitin nanofibrils (CNs) water suspension (2 wt % of concentration) was supplied by MAVI SUD (Latina, Italy). CNs represent the pure and polysaccharidic molecular portion of α-chitin obtained after elimination of the protein portion. These fibrils have an average size of 240 × 7 × 5 nanometers (nm) and a shape like thin needles [48]. The production process of chitin nano-fibrils patented by MAVI results in the formation of a stable aqueous suspension of nanofibrils containing 300 billion nano crystals per milliliter with the addition of sodium benzoate. This substance is an anti-MLD, added to the suspension to avoid the possible attack of mold and bacteria on the chitin [46].
- Two different typologies of calcium carbonate, commercialized by Omya SpA (Avenza, Italy), with different particle size distributions were used: Omyacarb 2-AV (named 2AV), Omya Smartfill 55-OM (named Smartfill). Hakuenka CC-R (named CCR) is commercialized by Shiraishi. 2AV has a micrometric particle size with a diameter value (relative to the maximum distribution curve, d_98%_) of 15 µm, and 38% of particles of diameter less than 2 µm. The average statistical diameter (d_50%_) is 2.6 µm with a specific weight of 2.7 g/cm^3^. Smartfill is fine ground and surface fatty acid treated calcium carbonate having 55% of particles with an average diameter <2 μm (bulk density: 1.1 g/mL). CCR is a precipitated nano-calcium carbonate coated with acids having an average particle size of 80 nm (specific weight of 2.6–2.7 g/cm^3^).

### 2.2. Characterization of Fillers

A Brunauer-Emmett-Teller (BET) analysis of the chitin nano-fibrils and of the three calcium carbonate fillers was carried out, with a micromeritics instrument-Gemini V analyzer (Micromeritics, Atlanta, GA, USA)—in order to determine the surface area, the total porosity and the number of particles per gram of the fillers. The measurement was performed with the same procedure used in the previous works of Coltelli et al. [47].

### 2.3. Blends Preparation and Torque Characterization

PLA/PBS blends preparation was carried out with a Haake Minilab II (Thermo Scientific Haake GmbH, Karlsruhe, Germany) co-rotating conical twin-screw extruder. Before processing the materials were dried in air circulated oven at 60 °C for at least 24 h. The molten materials were recovered in filaments for the subsequent tests. For each extrusion cycle, 6 g of PLA/PBS pellets, manually mixed with the other additives were fed through a little hopper into the mini-extruder. After the feeding, the molten material flowed in a closed circuit for 1 min at the end of which it was recovered. During this time, the torque was measured as a function of time, at least ten experimental measurements were performed for each blend compositions to guarantee the reliability and the consistency of the test. The final torque value is taken once that the melt stabilizes. The extrusion was carried out at 190 °C with a screw rotating speed of 110 rpm.

The compositions of the PLA/PBS blends investigated in this work, chosen considering the results of previous studies [44,47], are reported in Table 1.

To this purpose, the PLA/PBS ratio was maintained equal to 0.8 for all the formulations and also the ratio between ATBC and the polymeric matrix of PLA/PBS was kept constant and equal to 0.2. Furthermore, a fixed quantity (2 wt %) of Plastistrength (PST) was used for all blend compositions (except for F1 blend). The F3 formulation, according to previous studies [44,47] contained also a few percent (4 wt %) of micro-calcium carbonate (2AV). However, as mentioned, plasticizer migration was observed in these samples. Consequently, it has been decided to add a greater amount of calcium carbonate (7 wt %) and two other types of calcium carbonate with different particle size distributions to the blends to evaluate their effect on ATBC migration. F5 and F6 blends Smartfill and CCR were respectively added. The addition of chitin nanofibrils alone (F7 formulation) and coupled with the calcium carbonate particles, that showed better capability in hindering the plasticizer diffusion, was also investigated (F8 formulation).

For the preparation of the blends containing chitin nanofibrils (F7 and F8) PEG 6000 was used to achieve a better dispersion of the fibrils. The procedure adopted and the quantity of chitin nanofibrils chosen are described in the previous works of Coltelli et al. [47,48].

### 2.4. Melt Flow Rate

A CEAST Melt Flow Tester M20 (Instron, Canton, MA, USA) equipped with an encoder was used to investigate the melt flow behavior of the blends. The encoder, following the movement of the piston, acquires the melt volume rate (MVR) data. For each blend, three tests were carried out following the standard ISO 1133D [49]. According to the ISO procedure, the sample is simply preheated for 40 s at 190 °C after that, a weight of 2.160 kg is released on the piston and then, after 5 s, a blade cuts the spindle starting the real test. Every 3 s, MVR value is recorded by the encoder. The molten material, flowing through the capillary of specific diameter and length, is recovered and the MFR value is obtained. Before the test, the pelletized filaments of each polymer blend were dried in an air oven at 60 °C for one day.

### 2.5. Thermal Characterization by Differential Scanning Calorimetry (DSC)

Differential scanning calorimetry (DSC) measurements were performed with a DSC TA Instruments Q200 (TA Instruments, New Castle, UK), equipped with a RSC cooling system. Indium was used as the standard for calibration with aluminum hermetic pans. About 10 g of material was analyzed for each blend. Nitrogen was used as purge gas at a rate of 50 mL/min. The samples were heated at 10 °C/min from −40 °C to 220 °C. Only the first scan was considered to take into account the samples thermal history. Glass transition temperature (*T_g_*), melting temperature (*T_m_*), cold crystallization temperature (*T_cc_*), melting and cold crystallization enthalpies (Δ*H_m_* and Δ*H_cc_*) were determined by using the TA Universal Analysis software. In particular, the enthalpies of melting (Δ*H_m_*) and cold crystallization (Δ*H_cc_*) were determined from the corresponding peak areas in the heating thermograms; while the melting temperature (*T_m_*) and the cold crystallization temperature (*T_cc_*) were recorded at the maximum of the melting peak and at the minimum of the cold crystallization peak, respectively.

The crystallinity percentage (Xcc) of PLA in the blends was calculated as [50]

(1) $$X_{c}=\frac{\Delta H_{m,PLA}-\Delta H_{cc,PLA}}{\Delta H{{}^{\circ}}_{m,PLA}\cdot X_{PLA}}$$

where Δ*H_m,PLA_* and Δ*H_c,PLA_* are the melting enthalpy and the enthalpy of cold crystallization of PLA, *X_PLA_* is the weight fraction of PLA in the sample and Δ*H°_m,PLA_* is the melting enthalpy of the 100% crystalline PLA, equal to 93 J/g [51].

### 2.6. Tensile Test

The mechanical properties of the blends were evaluated by tensile tests carried out at room temperature. An INSTON 5500R universal testing machine (Canton, MA, USA), equipped with a 100 N load cell, was used. The machine, interfaced with a computer running a MERLIN software (INSTRON version 4.42 S/N-014733H), was assembled with compressed air grips (initial grip separation: 25 mm). The crosshead speed was set at 100 mm/min. The preparation of tensile specimens was carried out using the pelletized strains come out from the micro-compounder. The pellets were dried in an oven at 60 °C for 24 h to avoid the water uptake; subsequently, they were used for the film preparation by compression molding. The pelletized materials were pressed between two Teflon sheets at 180 °C for 1 min with a pressure of 3 tons, using a NOSELAB ATS manual laboratory heat press.

The mechanical tests were performed on an ISO 527-2 type A [52] dumbbell specimens obtained from the films cut with a Manual Cutting Press EP 08 (Elastocon, Brahmult, Sweden). At least 10 specimens were tested for each sample and the average values were reported.

### 2.7. Scanning Electron Microscopy Analysis (SEM)

Samples morphologies were investigated by scanning electron microscopy (SEM) with a FEI Quanta 450 FEG instrument (Thermo Fisher Scientific, Waltham, MA, USA). The micrographs of samples fractured with liquid nitrogen and sputtered with a layer of gold were collected. The metallic layer makes the surface electrically conductive, allowing the backscattered electrons to generate the images.

### 2.8. Migration Tests

Films prepared by compression molding (adopting the same procedure of film preparation described in Section 2.5) were used for the migration tests. To evaluate the weight loss of films due to the ATBC migration, three pieces of film for each formulation were put between two paper sheets. In this way, thanks to the capillarity forces related to the ATBC absorption from the paper sheets, the plasticizer is removed from the surface of the film ensuring a migration kinetics controlled by diffusion. The samples were kept in an oven at 60 °C (above T_g_) to make the test severe and to accelerate the migration process. Periodically, the films were weighed to estimate the weight loss as a function of time. The migration tests were stopped after 1500 h. For each formulation, the percentage weight loss of the film as a function of time was determined with the following relationship.

(2) $$\%wt\;loss=\frac{wt_{t,film}-wt_{t0,film}}{wt_{t0,film}}\cdot 100$$

where *wt_t,film_* is the film weight at the time *t* and *wt*_*t*0_, film is the film weight at the beginning of the test (*t* = 0). To separate the effect of calcium carbonate (used as an additive for hinders the plasticizer migration), from that of the loss of ATBC, was calculated the percentage of weight loss normalized respect to the amount of ATBC initially present in the film

(3) $$\%wt\;lost\;ATBC=\frac{\;lost\;ATBC\;mass}{initial\;ATBC\;mass}\cdot 100$$

In this way, it is possible to evaluate the films’ oiliness, thanks to the total weight loss of the film and appreciate the percentage by weight of ATBC that migrates from the film independently from the amount of plasticizer in the film.

## 3. Theoretical Analysis

For the prediction of the migration of monomers or additives, mathematical models are often used. In particular, the second Fick’s law is generally applied.

For a 3D system, the second Fick’s law is expressed as

(4) $$\frac{\partial c}{\partial t}=D\left[\frac{\partial^{2}c}{\partial x^{2}}+\frac{\partial^{2}c}{\partial y^{2}}+\frac{\partial^{2}c}{\partial z}\right]$$

If the diffusion is one-dimensional, that means that there is a gradient concentration only along one axis (for example x-axis), Equation (4) can be written in a simpler form (Equation (5)).

(5) $$\frac{\partial c}{\partial t}=D\frac{\partial^{2}c}{\partial x^{2}}$$

Commonly, the prediction of a substance migration through a polymer matrix above its glass transition temperature is described by the Fick’s second law based on one directional transfer (Equation (5)) [53]. General solutions of the diffusion equation can be adopted for a variety of initial and boundary conditions, provided the diffusion coefficient is constant. Crank and Vergnaud [54,55] proposed and classified different types of solutions for different geometries and boundary conditions. Taking into account the sample geometry adopted for the migration experiments, it is possible to apply one of the Crank’s solutions of the Fick’s second law. The equation used is reported below (Equation (6)) and it has been adopted successfully for plasticized polymeric systems [56,57]

(6) $$C_{x}=\frac{1}{2}C_{0}\left[\mathrm{erf}\left(\frac{h-x}{2\sqrt{D\cdot t}}\right)+\mathrm{erf}\left(\frac{h+x}{2\sqrt{D\cdot t}}\right)\right]$$

The parameters involved in Equation (3) are:

- *C_x_* (mg/cm^3^) that is the concentration of the chemical species that diffuses at a distance *x* from the center of the sample at the time *t*
- *C*_0_ (mg/cm^3^) is the starting concentration of the chemical species that diffuses at *t* = 0; thus, it will coincide with the initial concentration of the plasticizer present in the sample
- *D* is the diffusion coefficient (cm^2^/s)
- *h* (mm) is the sample thickness
- *erf* is the error function (where $erfz=\frac{2}{\pi }\int_{0}^{z}\exp\left(-\eta^{2}\right)d\eta$)

It can be observed that Equation (6) is symmetrical about *x* = 0 this means that the system can be cut in half by a plane at *x* = 0 without affecting the concentration distribution. It must be pointed out that the equation was obtained assuming that the migration occurred from the plasticized matrix to the same pure matrix, whereas our experiments were performed using paper, thus by removing the migrated plasticizer on the surface. Furthermore, if it is assumed that the plasticizer is initially distributed with a known concentration in the film and, if it is also supposed that the diffusion coefficient of the plasticizer in the polymer can be treated as a constant, a simple form of the Crank’s equation can be obtained by Equation (7) [54,58]

(7) $$M_{t}=2C_{P0}\sqrt{\frac{Dt}{\pi }}\;↔\;\frac{M_{t}}{C_{P0}}=\sqrt{D}\cdot 2\sqrt{\frac{t}{\pi }}$$

where *M_t_* (mg/cm^2^) is the total plasticizer lost from the film at time *t* (s), *C_p_*_0_ (mg/cm^3^) is the initial migrant concentration in the polymer and D (cm^2^/s) is the diffusion coefficient. Equation (7) also assumes that the film is sufficiently thick that the concentration of plasticizer at the mid-plane remains at its original value (*C_po_*) and this can be physically achieved if less than about of 15–20% of the plasticizer is lost [58]. If experimental migration data are available, the diffusion coefficient can be thus obtained by linear regression of the migration data as function of the square root of time (according to Equation (7)). Clearly, to adopt Equation (7), there must be sufficient data points available in the early part of the migration graph. Nevertheless, the principal criticism of this simplified equation is that the diffusion coefficient of plasticizer varies with the plasticizer concentration and thus this equation can be applied only for small amounts of plasticizer (low concentration). In fact, it is known in [59,60] that the diffusivity increases with the plasticizer concentration due to the increment of free volume and mobility of the polymer caused by the plasticizer addition. This dependence of diffusion with plasticizer concentration is well described by the exponential equation [54,61]

(8) $$D\left(C\right)=D_{c0}e^{\mathrm{aC}}$$

where *D_c_*_0_ is the zero-concentration diffusivity, and a is the plasticization coefficient related to the plasticizer efficiency. When the diffusivity is a function of the concentration, the differential equation (Equation (5)) is not linear. Normally for migration modeling the diffusion coefficient (*D*) is seen as concentration independent, which in most cases (if the system is not highly plasticized) can be acceptable [62].

However, it must be pointed out that two kinetics migration modes dominate the plasticizer loss: the diffusion mode (above mentioned) and the evaporation mode [23]. Considering only the one-dimensional problem in the x-direction (similarly to Equation (5)), the evaporation condition can be described by the mass balance [54,63]

(9) $$-D\left(C\right)\left(\frac{\partial C}{\partial x}\right)=F\left(C-C_{e}\right)$$

In Equation (9), the mass transfer related to the diffusion process (left term) is equated to the mass transfer of the plasticizer from the surface (right term). Obviously, evaporation can occur if the plasticizer concentration at the surface is greater than the concentration corresponding to the environment saturated with plasticizer (*C_e_*) [23].

**Figure 1:**
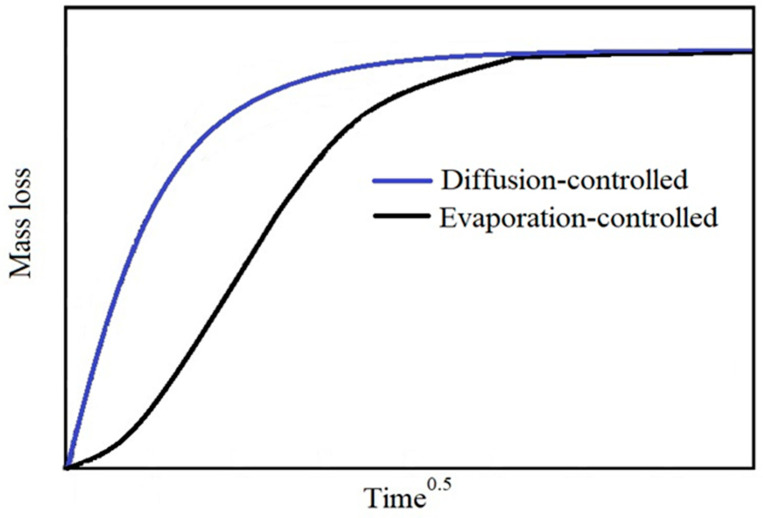
Examples of diffusion-controlled (blue line) and evaporation-controlled (black line) shape concentration profile.

Thus, the plasticizer migration will be affected by the coexistence of two phenomena: diffusion and evaporation; the overall rate of plasticizer loss will be determined by the slower process (diffusion- or evaporation-controlled). In the case of diffusion-controlled system, the evaporation rate will be faster than diffusion rate. On the other-hand, in the case of evaporation-controlled system the evaporation rate will be slower than diffusion rate and often this leads to a formation of a plasticizer film on the surface of the analyzed sample [64]. Generally, it is possible to understand if the system is diffusion- or evaporation-controlled, by the shape of the concentration profile obtained plotting the curves of mass loss versus the square root of the time (Figure 1).

The shape of concentration profile for an evaporation-controlled system, is flatter (S-shaped) if compared to the diffusion-controlled system [23].

It is evident that, for cases where the chosen plasticizer has a very high boiling point if compared to the temperature in which the material is currently used, the plasticizer will be accumulated on the external surfaces and it will form a thin film. The presence of this thin film of plasticizer that cannot easy evaporate due to the low temperature, limits the kinetic of plasticizer migration (the system will be evaporation-controlled). It is obvious that the concentration of plasticizer and the operative temperature will strongly influence the migration kinetics. Zhang et al. [65] demonstrated for a PLA system plasticized with acetyl triethyl citrate (ATC) that the ATC migration increases with the increasing of ambient temperature. The diffusion-controlled mechanism is activated for this system around 100–135 °C. Consequently, due to the similarity of the Zhang system with the polymeric system studied in this paper and also considering that the temperature adopted for the migration test (60 °C) is well below from the evaporation temperature of ATBC, it will be expected that the system will be evaporation-controlled.

The different film blends used for this work showed a plasticizer release phenomenon during the storage. From a practical point of view, it is difficult that the external film layer of plasticizer on the sample surface is not altered by external factors (presence of paper packaging which absorbs the plasticizer for example). Furthermore, there are some sectors (like cosmetics) in which a controlled release of these films is foreseen (for example in beauty masks). For these cases, it is evident how the diffusion process is the dominating mechanism.

**Figure 2:**
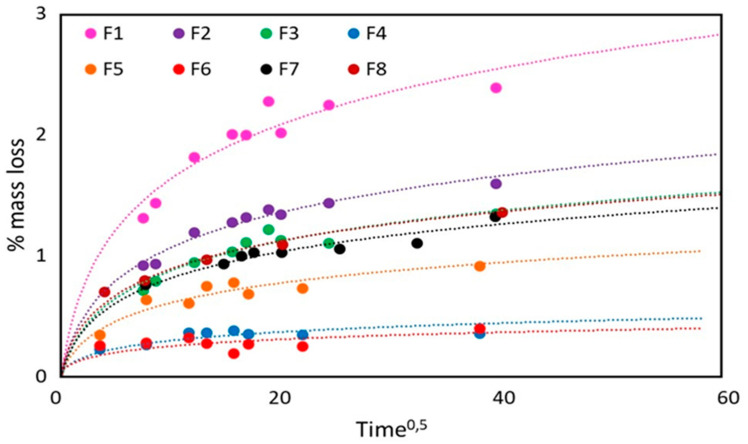
Concentration profile for all the compositions examined in this paper.

In this work, the migration studies were carried out with the goal of investigating the diffusion-controlled mechanism in order to evaluate the effect of micro and nano calcium carbonate addition on the plasticizer diffusion coefficient. Consequently, the experimental migration tests were also carried out in order to make the diffusion the kinetically controlling mechanism. The shape of concentration profile obtained for all the compositions examined (Figure 2), being diffusion shaped, confirmed the diffusion-controlling mechanism.

## 4. Results and Discussion

### 4.1. Migration Results and Determination of the Diffusion Coefficients

**Figure 3:**
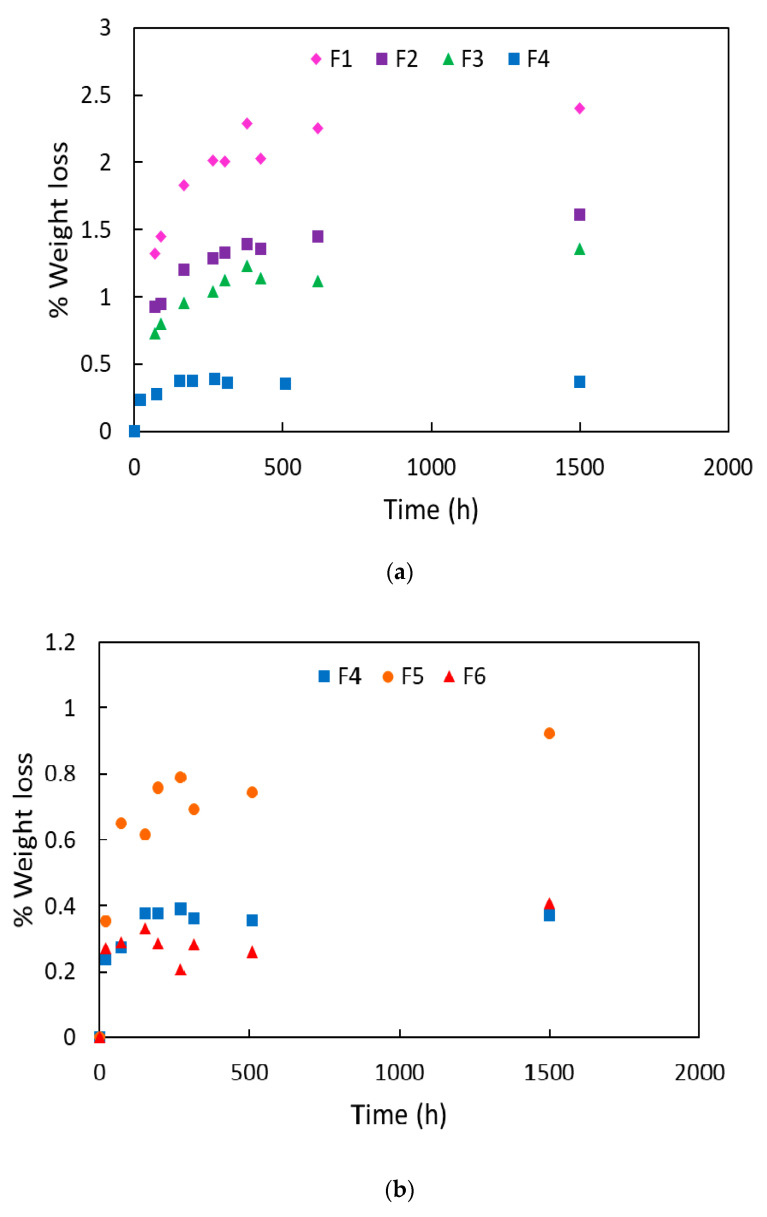

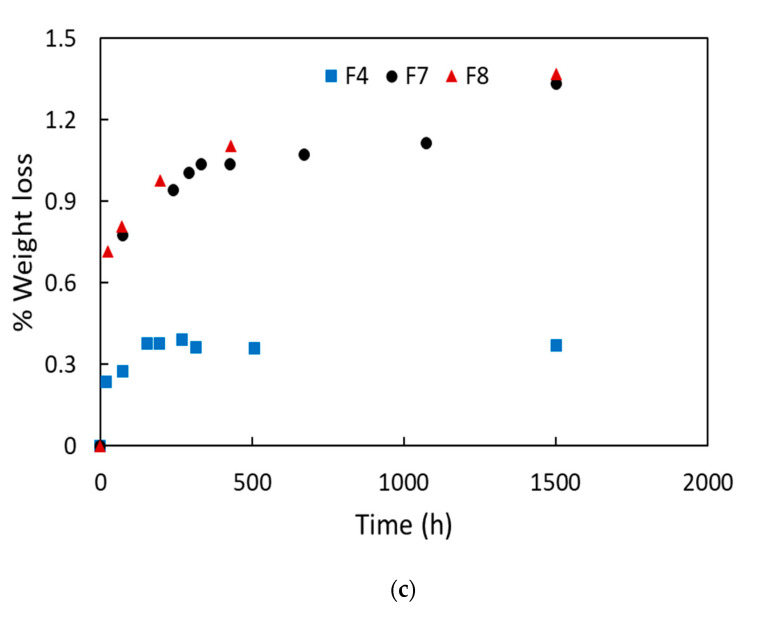
Weight loss percentage as function of the time for (**a**) F1, F2, F3 and F4 formulations, (**b**) F4 compared to F5, F6, (**c**) F4 compared to F7 and F8.

The trends of the weight loss percentage as function of the time are reported for all the blends in Figure 3. In Figure 3a, the loss percentages are reported for the reference formulations (F1, F2, and F3) compared to F4 where the quantity of micro-calcium carbonate (2AV) was incremented from 4 to 7 wt %. It can be noticed that F1 formulation has the higher weight loss if compared to F2. Both the formulations do not have micro-calcium carbonate; nevertheless, their plasticizer kinetic release is markedly different. This difference can be attributed to the addition of PST that, favoring the formation of intermolecular interaction [44], decreases the ATBC migration making more difficult the plasticizer diffusion. It can be observed that the addition of 4 wt % of micro-calcium carbonate (F3 blend), do not alter significantly the ATBC weight loss and only a slight slowing is observed if compered to F2. On the other hand, an increment from 4 wt % to 7 wt % significantly decreases the migration of ATBC and the weight loss percentage is reduced under the 0.5%. The major quantity of filler significantly slowed the plasticizer kinetic release. This can be ascribed to a major tortuosity path (generated by the increment of micro-calcium carbonate particles number) that the ATBC molecules must encounter. The plasticizer molecules have to follow a longer path in order to leave the polymeric structure [38].

**Figure 4:**
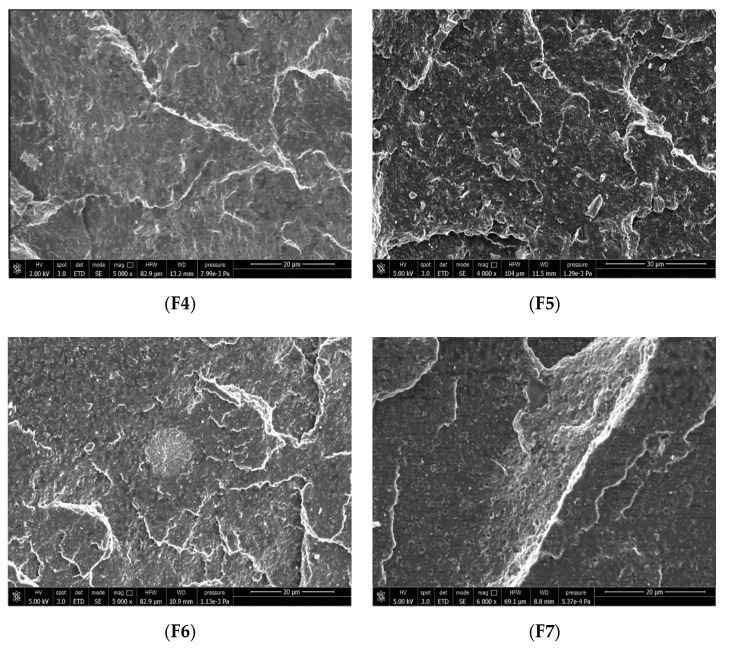

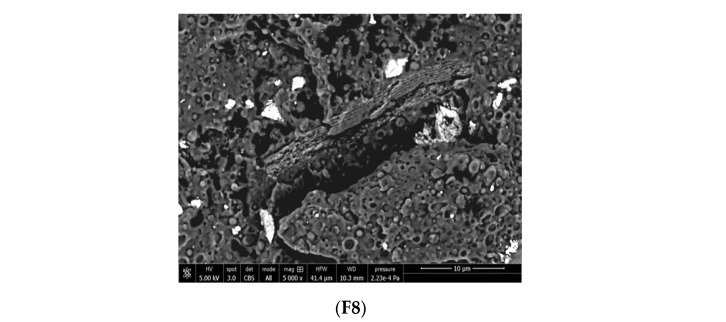
SEM micrographs of **F4**, **F5**, **F6**, **F7**, and **F8** formulations. The **F8** micrographs was obtained by backscattered electrons to better evidence CaCO_3_ particles (white).

The effect of the three different types of calcium carbonate particles (micro- and nano-, surface coated and not) are reported in Figure 3b. The best barrier properties to the ATBC migration are obtained with 2AV (F4). Moving from micro- to nano- calcium carbonate (F6 formulation), it would be expected a significant reduction of the plasticizer migration [35]. However, the data show a very similar trend with a slight decrease in the loss of ATBC. This result shows a poor efficiency of CCR (which also has a cost higher than Omycarb 2-AV). The explanation of this behavior can be ascribed to the presence of zones rich in agglomerates, as highlighted by the SEM analysis shown in Figure 4. It is likely that these surface coated nano-particles’ agglomeration is related to the manufacturing process of CCR. CCR, in fact, is a precipitated calcium carbonate where a water coating process was used. Generally the ‘wet’ processes have a minor surface coated area if compared to the ‘dry’ processes [66]. The coating in aqueous medium is different from solvent or dry coating, the process is controlled by micelle absorption followed by the micelle collapse into double or multiple layers during the drying stage. It has been demonstrated that for this process the monolayer coating is incomplete [67]. This incomplete coverage is responsible of the partially particles agglomeration. However, the presence of some nano-agglomerates cannot be the only reason for which the efficiency of the nano-metric fillers is not good enough. In fact, it must be considered that these nano-fillers are surface coated with fatty acids to improve their dispersion, reducing the surface tension between a hydrophobic and non-polar polymer and inorganic polar hydrophilic particles [68,69]. These surface agents, containing a polar group and a long aliphatic chain, can alter the ATBC absorption on the particles’ surface, worsening the hindering of ATBC migration. It is known in fact that the coating with fatty acids reduces the wettability of the particles to solvents such as water and n-decane [70]. Hence it is reasonable, on the basis of the results obtained, to hypothesize a negative chemical affinity (that reduces the ATBC absorption) between the ATBC and the aliphatic chains of fatty acids used for the surface coating. This conjecture can be reflected in the F5 formulation that contains surface-coated micro-calcium carbonate particles (Smartfill). The results achieved in the ATBC loss are worsened if compared to the not-surface-covered calcium carbonate particles, confirming the probable low absorption capacity of the calcium carbonate particles’ surface treated with fatty acids.

The results are worse where the coating with fatty acids is better; in fact, Smartfill is a ground calcium carbonate surface coated with a ‘dry’ process, hence it has a better surface coating if compared to CCR. Consequently, the ATBC absorption capacity of the calcium carbonate appears to be greatly worsened by this coating. The best results on the other hand are obtained with a calcium carbonate having no surface coverage.

Finally, in Figure 3c the effect of chitin nanofibrils alone and coupled to 2AV can be observed. It can be observed that chitin nanofibrils alone are capable of limiting the plasticizer release, and the migration level of ATBC is comparable to the F4 formulation. The coupling between chitin nanofibrils and 2AV does not show any type of synergy. The presence of agglomerates (that can be observed in the SEM of Figure 4) leads to a worsening of the final material barrier properties.

The quantitative results of the maximum weight loss, calculated according to Equations (2) and (3), are reported in Table 2 and confirms the trends of Figure 3.

The surficial area of the different fillers used in the present paper were determined by BET analysis and the results are reported in Table 3.

From Table 3, it can be noticed that CCR presents the higher Langmuir area among the calcium carbonates and a number of particles per gram two order of magnitude greater as a consequence of nanometric dimension. Moreover, chitin nanofibrils show a very high surficial area. The fillers with a higher surficial area should develop more interactions with the polymeric matrix.

However, it must be pointed out that a high surface area makes the aggregation of particles easier, confirming what has been observed by the SEM (Figure 4), where micrometric particles or agglomerates are revealed in all the samples. Chitin nanofibrils show the highest surficial area among the fillers, making them capable of agglomerating easily, resulting in micrometric bundles (Figure 4, F7 sample). This justifies the use of PEG to separate the fibrils as much as possible [47].

From the weight loss trends reported in Figure 3, two migration regimes can be observed: a first linear regime where a high quantity of plasticizer is lost in a short time and a second regime where the mass loss is lower and almost constant. The diffusion mechanism, as reported previously, is clearly influenced by the addition of the fillers (by their typology and quantity). However, another factor must be considered. The films, in fact, were maintained at 60 °C, slightly above the PLA glass transition temperature. At this temperature, the presence of compatible plasticizer (like ATBC) enhances the free volume of chains which induces molecular mobility. This reduces the Tg, but it can also favor the crystallization process [71]. The plasticizer goes only in the amorphous regions and if these amorphous regions decrease due to the crystallinity increment, the plasticizer migration will be accelerated. Considering that fillers can also act as nucleating agents (especially CaCO_3_) [40], film crystallization can become the main cause of ATBC loss. To better understand the role of the crystallization phenomena to the ATBC release, DSC analysis was performed considering only the first heating scan to take into account the real condition of the films produced by compression molding (i.e., considering their thermal history).

In Table 4 for all the formulations the values of glass transition temperature (*T_g_*), cold crystallization temperature (*T_cc_*), cold crystallization enthalpy (Δ*H_cc_*), melting temperature (*T_m_*), and melting enthalpy (Δ*H_m_*) are reported. Furthermore, the PLA percentage of crystallinity (*X_cc_*), calculated according to Equation (1), was reported. It can be observed that all the blends, which were held at 60 ° C, are well above their *T_g_*. Since these are plasticized films, their *T_g_*, compared to pure PLA, are shifted towards lower values. A marked decrement of PLA crystallinity from F1 to F2 formulation containing the PST can be observed. In literature, other authors confirmed that the PST addition decreases the number of crystals [72], this capability combined to the intermolecular interactions created by PST, limits considerably the plasticizer migration. The addition of 4 wt % of micrometric calcium carbonate (2AV) does not alter the crystallinity percentage initially present in the film. By adding larger quantities of filler (4–7 wt %), the starting crystalline content is significantly reduced independently of the type of calcium carbonate used. A greater quantity of filler not only hinders the diffusion process by increasing the tortuosity diffusion paths, but it also worsens the mobility of the polymeric chains and slows down the crystallization kinetics. It is known, in fact, that rigid fillers (such as calcium carbonate) can act as heterogeneous nucleation sites (nucleating agents) only if added in small quantities and if enough time is provided to the system for crystallizing [6]. Initially, the crystallinity degree does not influence the plasticizer migration that it is only affected by the type of filler used. At this purpose, the data confirm the negative role of the surface coating on the migration of ATBC making the micrometric 2AV the best choice in limiting the migration process.

On the other hand, F8 formulation shows a different crystallization behavior due to the interactions occurring between calcium carbonate particles and chitin nanofibrils. The not uniform distribution related to the formation of agglomerates facilitates the plasticizer migration and, at the same time, the PLA crystallization. The combination of these factors explains the major loss of ATBC that was encountered.

To better understand the migration mechanism of the plasticizer and if this is influenced during the time by an eventual crystallinity increment (due to the permanence of the sample at 60 °C for a long period), DSC analysis were performed at the end of the migration tests. The comparison was made only between the reference formulation F1, the formulation F2 containing the PST, the best filler calcium carbonate added formulation (F4) and for the chitin nanofibrils added formulation (F7).

**Figure 5:**
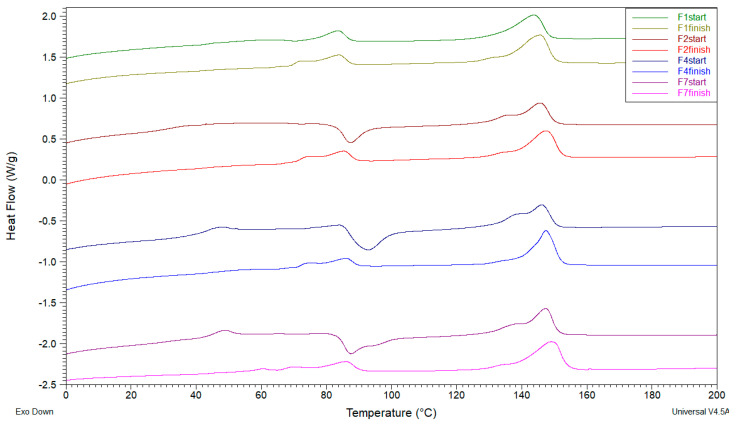
Comparison between thermograms before and after the migration test (first heating).

In Table 5, the DSC results at the end of the migration test for F1, F2, and F4 formulations are summarized. In this way, it is possible to verify how the crystallinity varies during the heat treatment at 60 °C. In Figure 5 the DSC thermograms before and after the migration test are also reported.

It can be observed that, over time, the crystallinity of the samples increases independently if there is the presence of PST or filler. This means that the migration of plasticizer over the time is influenced by the crystallinity increase that reduces the amorphous regions in where the plasticizer is situated and favors its migration. Anyway, it seems that independently from the additives, the system reaches the same value of PLA crystallinity (about 40%) that can be assumed as the final value reached over the time.

At this point, the diffusion coefficients for all formulations were evaluated adopting the linear diffusion coefficient obtained by regression of the migration data as a function of the square root of the time according to the simple form of the Crank’s equation (Equation (7)). According to Equation (7), by plotting the values of *M_t_*/*C_p_*_0_ (where Mt is the weight variation of the samples at time *t* per film area (mg/cm^2^) and *C_p_*_0_ is the initial density of the film (mg/cm^3^)) as a function of the time square root, the diffusion coefficient corresponds to the slope of the linear part of the curve. Clearly, this simplified equation, considering only the early stages of the migration where the trend is linear, does not take into account the crystallization phenomenon that may occur in in long periods and can induce a further plasticizer migration over the time. Consequently, the diffusion coefficients were also calculated by applying the not-simplified Crank’s solution of the second Fick’s law (Equation (6)). Considering that the films are very thin (0.025 cm), the concentration variation of the ATBC along the entire film thickness can be neglected thus x can be imposed equal to zero in Equation (6). The results obtained with Equation (6) make the calculation of the diffusion coefficients at various instants possible until the end of the migration tests (after 1500 h), considering in this way also the crystallization processes. An average diffusion coefficient weighted as a function of the plasticizer concentration was thus considered and compared with the diffusion coefficient obtained from Equation (7); the results are reported in Table 6.

The values of the diffusion coefficients calculated in the first stages by Equation (7) highlight the better efficiency of 2AV in limiting the plasticizer migration; in fact, for F4 formulation, the diffusion coefficient is lower of an order of magnitude. On the other hand, F8 shows the highest value of diffusion coefficients; the coexistence of the chitin nanofibrils and 2AV that forms agglomerates helps the ATBC diffusion leaving more routes for the plasticizer migration. Another good result is obtained by the diffusion coefficient of F6, containing the nano-metric CCR calcium carbonate. This result is coherent to what is observed from the ATBC mass loss. However, these diffusion coefficients consider only the initial diffusion mechanism where a very low value of crystallinity content was present for many formulations. As it was observed, the crystallinity increases up to 40% after 1500 h, consequently to consider the effects at longer times, the diffusion coefficients calculated by Equation (6) are more realistic.

First of all, it can be observed that considering the entire process, there is an increase of all diffusion coefficients and this confirms that the calculation only at the first stages lead to a D underestimation. A great worsening of the diffusion coefficients is registered for F1, F2, and F8 formulations coherently to what it was observed experimentally. In fact, these formulations lost a higher quantity of ATBC. The results confirm also that 4 wt % of 2AV is not a sufficient quantity to efficiently limit plasticizer migration; on the other hand, passing from 4 to 7 wt %, very good results are achieved. For longer times, the different behavior to the plasticizer migration for the three types of calcium carbonate emerges. The diffusion coefficients obtained confirm what was registered from the migration tests. In fact, it was observed that Smartfill was the worst filler in hindering the ATBC migration and, over long periods of time, its diffusion coefficient dramatically decreases. On the other hand, CCR seems to quite effectively limit plasticizer migration for long times with a slight decrement of its diffusion coefficient that passes from 1 × 10^−12^ cm^2^/s to 8.3·10^−11^ cm^2^/s. However, if we also compare these results with 2AV it can be observed that the improvement in the migration coefficients, and consequently in the ATBC loss, are not so evident to justify the use of this more expensive filler. Omycarb-2AV in fact, possess a *D* value of 8.2·10^−11^ cm^2^/s perfectly comparable with F6 diffusion coefficient. Noteworthy is the greatest difference in the diffusion coefficient calculated with the Equations (6) and (7), registered for the F6 formulation. Additionally, considering the crystallinity values over time (reported in Table 5), it can be observed that the crystallization kinetics for this type of formulation is slower therefore the higher ATBC release will occur in longer times. Equation (7) can therefore give a fairly truthful estimation of the diffusion coefficients only for those systems that quickly lose the plasticizer and for which there are no great differences of crystallinity over time.

As far as concern the formulation containing only the chitin nanofibrils (F7), a decrease in plasticizer migration should be observed. In fact, it is known in [73] that their addition improves the barrier properties. Effectively, the diffusion coefficient obtained is lower and confirms the mass loss results obtained. However, to contrast the ATBC migration, 2AV is still the most efficient.

(10) $$D=cost\cdot t^{-1}$$

**Figure 6:**
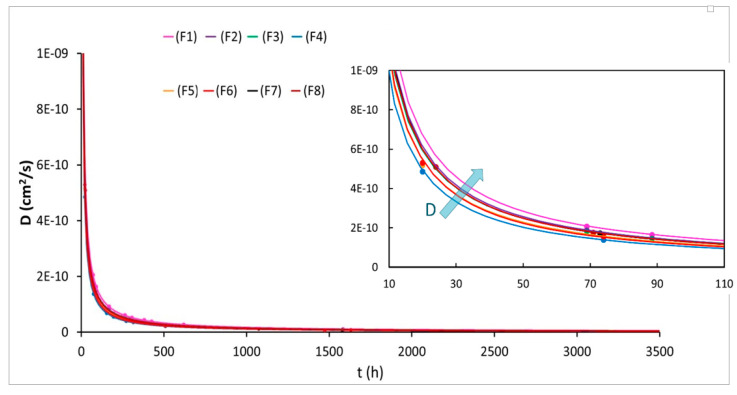
Trend of the diffusion coefficient over the time for all the formulation examined.

It can be observed from Figure 6 that the most significant variations of *D*, and therefore of the migration of the plasticizer, occur in the first 100 h—after the curves tend to flatten and the values of *D* tend to zero—meaning that the diffusion process finishes. The most flattened curve is that of the F4 formulation which will therefore have the lowest diffusion coefficient value over time and will therefore be able to release less plasticized content, further confirming the good efficiency of 2AV.

The rheological properties (torque, MFI, and MVR) properties were therefore evaluated and compared with those of the previous works [44,47].

The melt properties (MFR, MVR, and torque values) of each formulation are summarized in Table 7.

**Figure 7:**
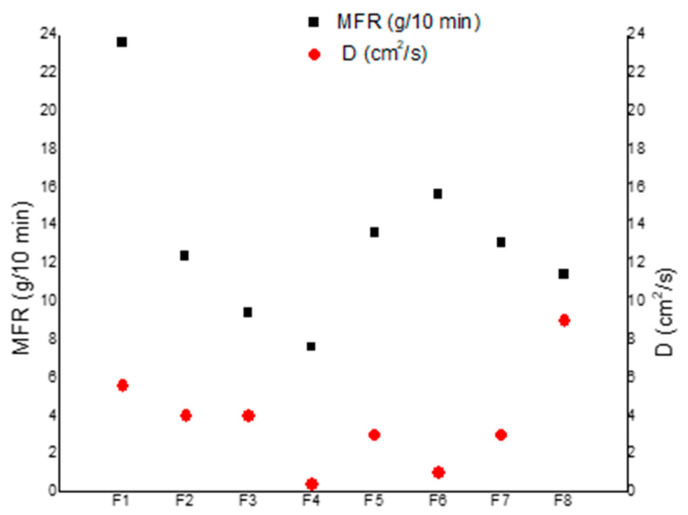
Comparison between MFR data and D calculated by Equation (6).

The addition of PST melt strength enhancer leads to an increment of torque (and in parallel to a decrement of MFR value) as it can be observed passing from F1 to F2 compositions. Clearly, the addition of a rigid filler (in this case micro calcium carbonate 2AV) leads to a marked decrement of the MFR value (F3 blend). As it can be expected, a further addition of micro-calcium carbonate (from 4 wt % to 7 wt %) further decreased the MFR from 9.4 to 7.6 (g/10 min) for F3 and F4 blends respectively. The MFR (similarly to MVR), affected by the molecular weight and by the interactions occurring in the melt material, can be correlated to the degree of disentanglement of the fluid blend [74]. The more the melt material is disentangled, the more the diffusion coefficient increases as calculated by Equation (6) (so considering only the starting part of the mass loss versus time trend) The material above its glass transition consists of a net of entangled macromolecules. A high molecular weight as well as efficient interactions contribute to increasing the degree of entanglement making more rigid the net and more difficult the diffusion of ATBC molecules. On the contrary, a high degree of disentanglement, due to lack of interactions with additives or fillers, as well as a decrease of molecular weight (cutting macromolecules), make the net more suitable for diffusion increasing D. The data of MFR, measured in the melt, and D decrease from F1 to F4. In fact, the degree of disentanglement decreases and D decreases accordingly (Figure 7).

However, in F5 using the same weight percent (7 wt %) of Smartfill with respect to F4 and CCR in F6, an increase in MFR was registered. It is likely that the surface coating of the calcium carbonate particles creates less friction in the molten matrix during the extrusion process. Moreover, as the removal of water during the drying of filler before the extrusion is more difficult for nanometric fillers, some chain scission due to water transesterification with polyester macromolecules can occur [75] resulting in shorter macromolecules. The latter effect influences the MFR confirming, this being the measurement done in the melt in dynamic conditions, so it is more dependent on the capability of macromolecules to flow. Both of these effects generate disentanglements that increase the value of D with respect to F4. In general, the observed variations in the Torque and MVR values are not so significant to alter the final processability independently of the filler used and also by the greater quantity of calcium carbonate added. Interestingly, F6 shows a very low value of D despite having the highest value of MFR. This is likely due to chain scission.

The occurring of chain scission was demonstrated by preparing a blend with the same composition of F6 but by previously drying better the nano-calcium carbonate, for two days at 60 °C at reduced pressure (50 KPa), and it was observed that the torque value was 77.0 ± 5.0 Ncm and the MFR value for this blend was 7.7 ± 0.6 cm^3^/10 min, in agreement with a higher molecular weight of the biopolyesters with respect to F6.

It can be observed that, for F6, the correspondent low value of *D* evidenced very good interactions, counterbalancing the decrease in molecular weight and reasonably achieved thanks to the nano-dimension of the filler (extended surficial area) significantly enhancing interactions despite a sub-optimal chemical affinity due to fatty acids and despite partial agglomeration. Chitin nanofibrils (F7) and micro-nanometric uncoated carbonate (F5), having an intermediate dimension between that of micro-carbonate and nano-carbonate, showed a similar behavior in agreement with the similar dimensions shown in phase morphology analysis of blends. In general, the analysis of the melt fluidity of the blends was fundamental to show that molecular weight can be another important parameter affecting the diffusion behaviors of polymeric materials.

### 4.2. Mechanical Characterization of Blends

After the evaluation of the different types of filler added to the ATBC migration, a general screening concerning the mechanical properties was carried out in order to evaluate if the mechanical properties (that were optimized in previous works [44,47]) were significantly altered by the greater amount of calcium carbonate added and by the different types of fillers used.

The variations of the tensile properties (yield stress (σ_y_), stress, and strain at break (σ_b_ and ε_b_)) are reported in Table 8.

**Figure 8:**
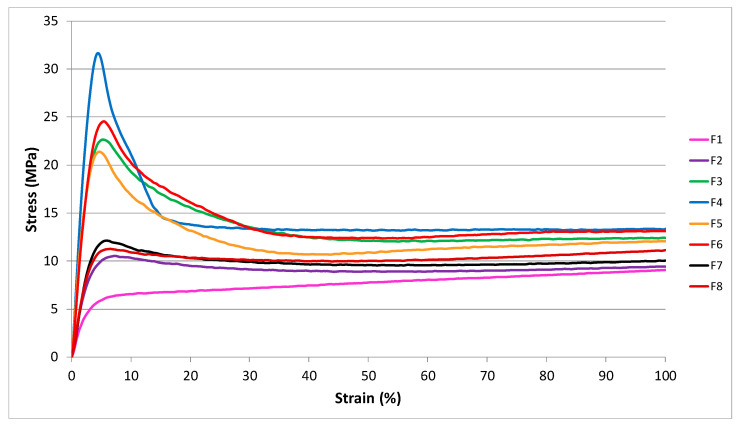
First part of the stress-strain curves for the formulations examined.

The increment up to 7 wt % of the different calcium carbonate fillers weakens the material. The greater quantity of rigid calcium carbonates added makes the final material more rigid, leading to a decrease of stress and elongation at break and to an increment of the yielding stress. However, comparing the final stress and strains values of the F3 formulation with those of F4, F5, and F6 it can be concluded that nevertheless this flexibility reduction the tensile properties of the plasticized PLA/PBS blends remains still good. The fillers introduced in these formulations, leads to a modification of the first part of the curve which assumes a typical trend of a more resistant system. In fact, it can be observed the capability of PST and fillers to increase the yield stress compared to the F1 formulation, which exhibits a behavior similar to an elastomer (Figure 8). The addition of an uncoated micrometric carbonate such as 2AV, capable of dispersing adequately during extrusion, ensures a yield value higher than other types of carbonates.

The combination of 2AV with chitin nanofibrils does not lead to an improvement of the tensile properties, as can be observed in F8 compared to F4, especially as regards the elongation at break. For F8 formulation, the yield stress is very similar to the result obtained with chitin nanofibrils alone. On the other hand, the comparison with the F4 formulation shows a marked decrease in the yield stress. The nanofibrils seem to not allow calcium carbonate to reinforce the formulation. Standing between the carbonate particles and the polymer matrix, they cause a reduction of the filler-matrix adhesion as it can be observed from the SEM image (Figure 4) where the micrometric calcium carbonate was found both in the matrix and near the areas containing nanofibrils.

## 5. Conclusions

On the basis of previous studies, the plasticized poly(lactic acid) (PLA)/poly(butylene succinate) (PBS)-based films containing chitin nanofibrils (CNs), calcium carbonate, and a small percentage of a commercial melt strength enhancer, have been investigated from the point of view of the plasticizer migration. It was observed that, during storage, these films lost a significant amount of plasticizer. The effect of the addition of different types of calcium carbonate (nanometric and micrometric, surface coated and not) and chitin nanofibrils was investigated with the purpose of reducing or controlling the plasticizer migration. The Crank’s solution of the Fick’s second law was adopted to obtain the quantative values of diffusion coefficients. In addition, the evolution of the crystallinity that can induce the plasticizer migration was considered.

The results showed that the not surface coated calcium carbonate (2AV) is the more effective in hindering the plasticizer migration. Micrometric calcium carbonate reduced significantly the ATBC migration but at the same time did not lead to a significant change in the processability and mechanical properties of the already optimized formulations.

Regarding the surface coated calcium carbonate, the surface coating (generally made with fatty acids) probably limits the ATBC absorption on the calcium carbonate particles surfaces, leading to worse results in ATBC migration. Moreover, the occurrence of chain scission due to difficulties in removing humidity from nanofiller before blending determined a decrease in molecular weight, resulting in macromolecular net disentanglement that allowed a better diffusion of ATBC, as concluded by studying the fluidity in the melt of blends. For these reasons, the migration control is not significantly improved passing from micro- to nano- calcium carbonate particles, despite the increased surficial area of fillers. From a point of view of cost, reduction of the plasticizer migration, and quantity added, the nanometric calcium carbonate particles are less efficient and this is ascribable to their agglomeration tendency and high hygroscopicity.

Chitin nanofibrils alone can also influence the plasticizer migration thanks to their capability of hindering the plasticizer diffusion, slowing but not efficiently limiting the migration, and thus suggesting new potentialities of such nano additives in films or products with a controlled release.

The coupling of chitin nanofibrils and 2AV did not lead to significant results. Negative effects have been encountered probably related to the synergistic agglomeration tendency of chitin nanofibrils and calcium carbonate.

The crystallinity reached from samples after migration test seems not affected by the type of filler added and about 40% of PLA crystallinity was obtained. Clearly, this marked crystallinity increment over time affects the plasticizer migration due to the decreasing portion of mobile amorphous fraction in which the plasticizer is located.

In general, the analysis of all the parameters affecting migration of plasticizers suggests promising strategies to better exploit the properties of inorganic micro and nano particles, as well as natural nanofibrils in biocomposites.

## Figures and Tables

**Table 1:** Blends name and composition.

Blends Name	PLA (wt %)	PBS (wt %)	ATBC (wt %)	PST (wt %)	CaCO_3_ (wt %)	PEG6000 (wt %)	NC (wt %)
**F1**	63	17	20	-	-	-	-
**F2**	62	16	20	2	-	-	-
**F3**	59	15	20	2	4 (2AV)	-	-
**F4**	59	15	17	2	7 (2AV)	-	-
**F5**	59	15	17	2	7 (Smartfill)	-	-
**F6**	59	15	17	2	7 (CCR)	-	-
**F7**	61	15	18	2	-	2	2
**F8**	57.5	14.5	15	2	7 (2AV)	2	2

**Table 2:** Film weight loss and ATBC lost, calculated according to Equations (2) and (3), for all film formulations.

Blends Name	Weight Loss (wt %)	Lost ATBC (wt %)
**F1**	2.40 ± 0.31	12.01 ± 1.53
**F2**	1.61 ± 0.01	8.05 ± 0.08
**F3**	1.36 ± 0.19	6.78 ± 0.95
**F4**	0.39 ± 0.15	2.29 ± 0.08
**F5**	0.92 ± 0.14	5.44 ± 0.83
**F6**	0.22 ± 0.07	1.31 ± 0.43
**F7**	1.33 ± 0.43	7.40 ± 2.38
**F8**	1.37 ± 0.16	9.12 ± 1.06

**Table 3:** Surficial areas, porosity, and number of particles per gram of the four fillers used in this paper.

Samples	Langmuir Area (m^2^/g)	BET (m^2^/g)	Total Pore Volume (cm^3^/g)	Number of Particles Per Gram
2AV	4.3094	2.7126	0.0013	1.3 × 10^12^
Smartfill	6.1180	3.7659	0.0018	3.5 × 10^12^
CCR	32.1068	18.4610	0.0085	4.0 × 10^14^
NC	61.7235	39.1443	0.0194	4.1 × 10^13^

**Table 4:** Results of differential scanning calorimetry analysis (first heating).

Blends	*T_g_* (°C)	*T_cc_* (°C)	Δ*H_CC_* (J/g)	*T_m_* (°C)	Δ*H_m_* (J/g)	*X_C_* (%)
F1	43.6	96.4	3.05	143.7	18.84	27
F2	31.7	86.7	12.97	145.3	19.94	12
F3	30.3	85.4	13.30	144.8	19.19	11
F4	44	92.9	17.56	145.9	20.44	5
F5	36.2	90	16.93	146.5	18.6	3
F6	44.1	93.3	18.37	147.9	19.92	3
F7	46.4	87.5	19.31	143.4	20.57	2
F8	43.5	85.7	11.97	145.5	18.82	13

**Table 5:** DSC results for F1, F2, F4, and F7 formulations at the beginning and at the end of the migration test (first heating).

Blends	*T_g_* (°C)	*T_cc_* (°C)	Δ*H_CC_* (J/g)	*T_m_* (°C)	Δ*H_m_* (J/g)	*X_C_* (%)
F1_start_	43.6	96.4	3	143.7	18.8	27
F1_finish_	42.4	-	-	145.6	23.5	40
F2_start_	42.7	86.7	12.9	145.3	19.9	12
F2_finish_	43.5	-	-	146.8	21.8	38
F4_start_	44	92.9	17.5	145.9	20.4	5
F4_finish_	43.8	-	-	147.4	21.9	40
F7_start_	46.4	87.5	19.31	143.4	20.57	2
F7_finish_	48.8	-	-	149.1	21.84	38.5

**Table 6:** Diffusion coefficient calculated according to Equation (7) and to Equation (6).

Blends	D (cm^2^/s) Equation (7)	D (cm^2^/s) Equation (6) Weight Averaged
F1	5.6 × 10^−12^	1.3 × 10^−10^
F2	4 × 10^−12^	1.2 × 10^−10^
F3	4 × 10^−12^	1.1 × 10^−10^
F4	4 × 10^−13^	8.2 × 10^−11^
F5	3 × 10^−12^	9.8 × 10^−11^
F6	1 × 10^−12^	8.3 × 10^−11^
F7	3 × 10^−12^	9.7 × 10^−11^
F8	9 × 10^−12^	1.3 × 10^−10^

**Table 7:** Torque, MVR, and MFR values of the prepared blends.

Blends	Torque (N∙cm)	MVR (cm^3^/10 min)	MFR (g/10 min)
F1	67.8 ± 5.4	22.5 ± 2.0	23.6 ± 2.1
F2	72.8 ± 6.0	11.8 ± 0.9	12.4 ± 0.9
F3	73.4 ± 9.8	8.7 ± 0.6	9.4 ± 0.6
F4	83.4 ± 4.0	8.4 ± 2.7	7.6 ± 2.4
F5	74.1 ± 5.4	12.0 ± 1.9	13.6 ± 2.2
F6	66.4 ± 5.9	14.1 ± 1.9	15.6 ± 2.1
F7	63.3 ± 3.6	11.8 ± 1.1	13.1 ± 1.3
F8	73.5 ± 5.2	10.0 ± 1.3	11.4 ± 1.5

**Table 8:** Tensile properties of the films obtained from each blend.

Blends	σ_b_ (MPa)	ε_b_ (%)	σ_y_ (MPa)
**F1**	31.8 ± 1.4	572.7 ± 20.7	-
**F2**	33.0 ± 1.2	554.2 ± 12.3	10.2 ± 0.7
**F3**	32.5 ± 1.6	543.7 ± 29.8	23.3 ± 1.9
**F4**	29.3 ± 3.4	512.5 ± 13.8	31.1 ± 2.2
**F5**	29.0 ± 1.2	491.4 ± 25.9	20.7 ± 2.4
**F6**	28.4 ± 1.8	507.6 ± 13.9	24.5 ± 1.7
**F7**	25.5 ± 1.3	421.9 ± 25.1	11.6 ± 0.7
**F8**	25.5 ± 1.0	400.1 ± 21.9	10.8 ± 1.8
